# Insulin-like growth factors and insulin control a multifunctional signalling network of significant importance in cancer

**DOI:** 10.1038/sj.bjc.6605932

**Published:** 2010-10-05

**Authors:** P Massoner, M Ladurner-Rennau, I E Eder, H Klocker

**Affiliations:** 1 Section of Experimental Urology, Department of Urology, Innsbruck Medical University, Anichstr. 35, Innsbruck 6020, Austria

**Keywords:** cancer therapy, IGF, insulin

## Abstract

Insulin-like growth factor (IGF) and insulin (INS) proteins regulate key cellular functions through a complex interacting multi-component molecular network, known as the IGF/INS axis. We describe how dynamic and multilayer interactions give rise to the multifunctional role of the IGF/INS axis. Furthermore, we summarise the importance of the regulatory IGF/INS network in cancer, and discuss the possibilities and limitations of therapies targeting the IGF/INS axis with reference to ongoing clinical trials concerning the blockage of IGF1R in several types of cancer.

## IGF/INS Proteins Control an Interacting Multifunctional Regulatory Network

Insulin-like growth factor (IGF) and insulin (INS) proteins orchestrate a regulatory network of multiple components with dynamic interactions, named herein the IGF/INS axis. Insulin-like growth factor/INS proteins regulate crucial functions in tissue homeostasis and malignant growth, including proliferation, survival, tissue homeostasis, differentiation, energy supply, energy consumption and cellular metabolism. The main sources of circulating IGFs and INS are the liver and *β*-cells of the islets of Langerhans of the pancreas, respectively. In addition, there are local productions of IGFs and INS in many cells and tissues.

The IGF/INS axis is composed of the receptor ligands IGF1, IGF2 and INS, high-affinity (IGFBP) and low-affinity (IGFBP-rP) IGF-binding proteins that exert modulatory functions, and the receptors IGF1 receptor (IGF1R), IGF2 receptor (IGF2R), INS receptor (INSR) and INSR-related receptor (IRR). Upon activation, IGF/INSRs initiate a complex intracellular signaling network across the major signaling pathways PI3K–AKT and RAS–RAF–MAPK, and thus control a variety of cellular processes in normal physiology and pathophysiology.

## Multifunctional Effects in Physiology and Pathophysiology

To date, 15 molecular functions and 29 biological processes have been linked to IGF1R, whereas 22 molecular functions and 47 biological processes have been linked to INSR in the gene ontology database (http://www.geneontology.org; definitions: molecular function, the elemental activities of a gene product at the molecular level; biological process, operations or sets of molecular events with a defined beginning and end, pertinent to the functioning of integrated living units: cells, tissues, organs and organisms. Query August 2010). This shows that the IGF/INS axis is a multifunctional protein family and we can provide only an outline of the best-characterised IGF/INS effects. The reader should keep in mind the fact that the IGF/INS multifunctional network will have additional functions, depending on the cell type and the cellular context.

IGF/INS proteins are expressed ubiquitously, but in different ratios and amounts, and exert auto-, para- and endocrine biological effects in a variety of tissues and cells: (1) IGF/INS growth factors act as growth hormones and regulate the growth of human tissues and cells (Pollak, 2008). Severe growth retardations were found in humans with defects in *IGF/INS* genes (Ohlsson *et al*, 2009), whereas studies in knockout mice have confirmed the pivotal role of the IGF/INS axis in normal growth (Ohlsson *et al*, 2009). Regulation of the life span has been associated with IGF expression levels (Holzenberger *et al*, 2003). (2) The IGF/INS axis is required to maintain tissue homeostasis (Sutherland *et al*, 2008) and a differentiated phenotype in normal tissue (Belfiore *et al*, 2009). (3) The IGF/INS network influences the balance between apoptosis and survival. IGFs are anti-apoptotic and pro-survival factors—effects that are of major importance in the emergence and progression of cancer (LeRoith and Roberts, 2003; Pollak, 2008). (4) A further important function of the IGF/INS network is in metabolism, where INS is a key regulator controlling cellular glucose, amino-acid and fatty-acid uptake, as well as glycogen, lipid and protein synthesis and a variety of other related metabolic processes (Saltiel and Kahn, 2001). (5) The IGF/INS network is also involved in angiogenesis, cell adhesion, migration and wound healing (LeRoith and Roberts, 2003) and (6) (less known) exerts multiple effects in the brain. It also influences mammalian behavior and memory (Broughton and Partridge, 2009; Ohlsson *et al*, 2009).

**Figure 1:**
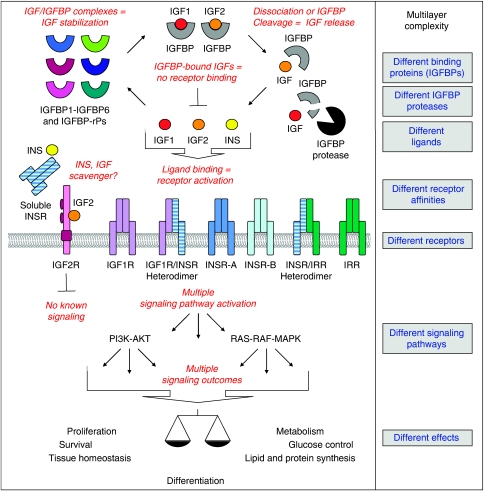
The IGF/INS axis is a complex multilayer interacting molecular network with multiple effects.

We do not entirely know how the IGF/INS axis mediates these multiple functions, but experimental data have shown that dynamic and multilayer IGF/INS interactions, and cross talks of the IGF/INS axis to other receptor tyrosine kinase pathways, such as the epidermal growth factor receptor (EGFR) pathway, generate variable IGF/INS signals in different cell types and tissues. The different layers of diversity of signalling that give rise to the multiple functions of the IGF/INS axis are schematically shown in Figure 1 and explained in the following chapters. The variety of cellular responses to the IGF/INS signal depend, on one hand, on the availability of growth factors and the ratios of the receptors and signalling molecules, and, on the other hand, on the cell and tissue type and the tissue microenvironment.

## IGF/INSRs – complexity through heterodimer formation

IGF/INSRs include IGF1R, IGF2R, INSR, the latter existing in two different isoforms, namely INSR-A and INSR-B, and IRR. IGF1R, INSR and IRR are composed of an extracellular ligand-binding domain and an intracellular protein kinase domain. Their structural similarity permits the formation of heterodimer receptors formed by subunits of different receptor proteins, such as IGF1R/INSR and INSR/IRR heterodimers (Jui *et al*, 1996; Pandini *et al*, 2002). Heterodimers are spontaneously formed when the different receptors are expressed, and are the most abundant receptor subtype in many tissues.

The receptors bind IGF and INS ligands with different affinities: Ranking from high to low and very low affinity, IGF1R binds IGF1, IGF2 and INS; IGF2R binds IGF2 and other ligands, such as mannose-6-phosphate, IGF1 and INS; and INSR binds INS, IGF2 and IGF1 (Pandini *et al*, 2002; Ghosh *et al*, 2003; Belfiore *et al*, 2009). INSR-A has a higher IGF2 affinity than INSR-B (Belfiore *et al*, 2009). IRR is an orphan receptor with unknown binding ligand that participates in signal transduction as a heterodimerisation partner of the ligand-binding family members (Zhang and Roth, 1992). Evidently, the subunit composition of receptors determines their affinity for ligands. For instance, IGF1R/INSR-A heterodimers possess a high affinity for IGF2 (Pandini *et al*, 2002; Belfiore *et al*, 2009).

It is of note that IGF1R/EGFR heterodimers have also been characterised (Morgillo *et al*, 2006), but their abundance and ligand affinity remain unclear. A few reports describe the occurrence of a soluble INSR that is secreted from cultured human cells (Papa *et al*, 1993) and appears in human plasma (Pezzino *et al*, 1992). This soluble INSR was shown to bind INS (Papa *et al*, 1993); its IGF binding has not yet been investigated.

Taken together, the classical view that IGFs bind and activate IGF1R and INS activates INSR greatly simplifies the biological situation in which IGF/INS-responsive receptors constitute a complex interacting receptor network. Depending on the availability of IGF/INS ligands and the ratios of IGF/INS-responsive receptors, IGFs can also activate INSR and, conversely, INS activates IGF1R.

## IGF binding proteins modulate receptor ligands

IGF1 and IGF2 are bound by six high-affinity binding proteins, named IGFBP from IGFBP1 to IGFBP6, and several low-affinity binding proteins, known as IGFBP-related proteins (IGFBP-rPs). IGFBPs and IGFBP-rPs have different degrees of IGF affinity: IGFBP1–IGFBP5 have higher affinities for IGF1, whereas IGFBP6 has a higher affinity for IGF2 (Firth and Baxter, 2002). Moreover, IGFBPs and IGFBP-rPs have the ability to bind INS, but with a very low affinity (Hwa *et al*, 1999).

IGF/IGFBP complexes exert two principal functions: first, they stabilise IGFs and protect them from degradation, thus extending their lifespan from a few minutes to several hours. Second, they inhibit the binding of IGFs to their receptors. Therefore, only IGFs that are released from IGFBPs by dissociation or protease-mediated IGFBP cleavage can induce IGF signals (reviewed by Hwa *et al*, 1999; Firth and Baxter, 2002).

In general, these IGF-related effects are similar and, in part, redundant for all IGFBPs. However, different IGFBP affinities, structures and post-translational modifications (Hwa *et al*, 1999), as well as the presence of specific IGFBP proteases (Sadowski *et al*, 2003) and IGFBP-binding extracellular matrix components (Nam *et al*, 2002), influence the effects of IGFBPs and permit fine-tuning of IGF/IGFBP interactions in different tissues and tissue areas. IGFBP binding to IGFs is usually inhibitory for receptor activation, but under certain circumstances IGFBPs can promote IGF signalling and may have pro-oncogenic effects. IGFBPs stabilise and slowly release IGFs for receptor interactions, thereby preventing receptor downregulation by high IGF exposure. Thus, they promote a prolonged and constant receptor activation (Firth and Baxter, 2002).

Beside their functions in IGF complexes, IGFBPs and IGFBP-rPs exert effects that are independent of IGF binding or signalling and specific for single IGFBPs and IGFBP-rPs. For instance, IGFBP-3 was found to act as an anti-cancer protein by inhibiting proliferation, adhesion and motility by IGF-independent mechanisms (Firth and Baxter, 2002). IGFBP-rP1 (IGFBP7) was described to have a central role in BRAF-mediated senescence and apoptosis in melanoma cells (Wajapeyee *et al*, 2008). Taken together, IGF/IGFBP complexes are tightly regulated and highly dynamic extracellular complexes that modulate the IGF/INS signal by influencing IGF/INS ratios, growth factor stabilities, receptor binding and duration of receptor activation.

## IGF/INSR downstream signalling pathways

IGF1R, INSR and IRR are tyrosine kinase receptors that initiate an intracellular signal upon receptor activation. The signal transduction is mediated by both homo- and heterodimer receptors. Not only in homodimers but also in heterodimers both parts of the receptor can be activated upon ligand binding. Thus, IGF1R/INSR heterodimers activate IGF1R and INSR signalling (Belfiore *et al*, 2009), and INSR/IRR heterodimers activate INSR, and also the orphan IRR (Zhang and Roth, 1992).

IGF2R has no apparent intracellular signal and is believed to act as a scavenger receptor for IGFs. The main function of IGF2R is related to other pathways rather than IGF signalling: IGF2R is required for the delivery of acid hydrolases from the Golgi network to endosomes and lysosomes (Ghosh *et al*, 2003). The role of the soluble INSR in the IGF/INS axis is yet to be investigated, but the soluble INSR may also well function as a scavenger receptor, thereby reducing the extracellular availability of INS.

**Figure 2:**
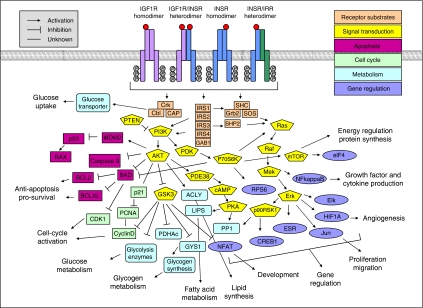
IGF/INS ligand binding, to one or both receptor monomers, leads to the activation of IGF1R, INSR and IRR, which activate a complex signalling network across the two major signalling pathways PI3K–AKT and RAS–RAF–MAPK (shown here in parts (for detailed signalling networks see Kyoto Encyclopedia of Genes and Genomes pathway database (http://www.genome.jp/keg) and GeneGO pathway analysis database (http://www.genego.com)). The IGF/INS axis regulates multiple functions in normal physiology and pathophysiology. IGF1R and INSR have been reported to act by means of the same pathways. The signal is influenced by different binding velocities, reaction times, activities, expression levels and sub-cellular locations of signalling molecules. Selective substrate specificities for IGF1R and INSR probably also exist.

IGF/INS signal transduction may be summarised as follows: IGF/INS binding to IGF1R and INSR homo- and heterodimers induces receptor clustering, autophosphorylation, and stimulation of receptor tyrosine kinase activity, leading to the recruitment and phosphorylation of IRS-1 and Shc, which activate (directly or by association with Grb-2/SOS) the two signalling pathways PI3K–AKT and RAS–RAF–MAPK. These pathways, in turn, initiate a variety of intracellular signalling cascades that have multiple effects on gene regulation and protein expression, activation and translocation (LeRoith and Roberts, 2003). However, the IGF/INS signal is more complex than this simplified view suggests: a variety of other substrates for IGF1R and INSR, such as other IRSs, Gab1, Cbl, APS, SHP2, Fyn and Csk, have been described (Belfiore *et al*, 2009). Availability, location and ratios of these receptor substrates significantly influence cellular responses upon IGF1R and INSR activation. Some of the main signalling molecules, such as PI3K, appear in different isoforms that have different activation and effector properties (Saltiel and Kahn, 2001). Several cross-talks of the IGF/INS signalling to other pathways have been reported, which are mediated by interactions of IGF1R and INSR with major signalling molecules of other molecular pathways, such as SOCS interactions influencing the JAK–STAT pathway (Dey *et al*, 1998), or interactions with other receptors, such as IGF1R/EGFR heterodimers (Morgillo *et al*, 2006). Thus, IGFs and INS activate, by means of IGF1R and INSR, a highly complex intracellular signaling network (Figure 2).

IGF1R and INSR have been reported to act through the same pathways. However, subtle differences in the recruitment of docking proteins and intracellular mediators cause a fine-tuned signalling outcome that permits selective IGF1R or INSR signal transduction (Pandini *et al*, 2002). These differences arise not only by different substrate specificities for IGF1R and INSR, which probably do exist, but are also mediated by different binding velocities, reaction times, activities, expression levels and sub-cellular locations of signalling molecules (Sasaoka *et al*, 1996; Biedi *et al*, 2003).

In summary, IGF/INS ligand binding leads to the activation of IGF1R, INSR and IRR, which activate a complex signalling network across the two major signalling pathways PI3K–AKT and RAS–RAF–MAPK. With regard to the close and reciprocal interactions of INS and IGF signalling in the IGF/INS network, the activation of a single pathway (such as IGF signalling) with no activation of the other pathway (such as INS signalling) appears to be unlikely. However, fine-tuning of ligand availability, receptor ratios, signalling molecule expression and localisation alter the signal, thus allowing predominant activation of IGF/IGF1R or INS/INSR signals in different cells and tissues.

## The IGF/INS axis and cancer

Evasion from apoptosis and a limitless replicative potential are two hallmarks of cancer (Hanahan and Weinberg, 2000). Thus, growth and survival pathways have emerged as appealing targets for cancer therapy. As the IGF axis is a central regulator of growth and survival, therapeutic strategies focusing on the blockage of IGF1R and IGF signalling are currently being investigated for several types of cancer in a large number of clinical trials. In the following we will discuss the rationale underlying these trials, their power and their limitations in respect of the IGF/INS network.

Cumulative evidence has been obtained concerning the role of the IGF/INS network in the emergence and progression of cancer. Genetic studies showed that the risk of cancer is influenced by *IGF/INS* gene variants, and cancer-associated somatic copy number variations are found in *IGF/INS* gene regions. Recent examples are an *IGF1* polymorphism associated with non-small-cell lung cancer (Zhang *et al*, 2010), an *IGF2R* polymorphism influencing the risk of oesophageal and gastric cancer (Hoyo *et al*, 2009), and the detection of a cancer-associated somatic copy number amplification in the *IGF1R* gene region in a pooled analysis of 26 cancer types, each represented by more than 20 specimens (Beroukhim *et al*, 2010). Epidemiological studies also revealed that high IGF1 serum concentrations are associated with prostate, colorectal and breast cancer (Renehan *et al*, 2004). Epigenetic, expression and protein analyses have demonstrated alterations of IGF/INS expression and protein levels in cancer tissues. Increased expression of IGF2 caused by loss of genomic *IGF2* imprinting (genomic imprinting: only one allele of the gene is active, depending on the parental origin) was registered for cervical cancer, choriocarcinoma, colorectal cancer, hepatoblastoma, lung cancer, rhabdomyosarcoma, pediatric testicular cancer and Wilms’ tumours (Ross *et al*, 1999; Chao and D’Amore, 2008). IGF1R was found to be essential for oncogenic transformation in certain cellular systems. Mouse fibroblasts lacking IGF1R cannot be transformed by known oncogenes, such as SV40 T antigen, papillomavirus E5 and overexpression of Ras (Sell *et al*, 1993). Overexpression of a constitutively activated IGF1R was shown to be sufficient to cause mammary epithelial cell transformation in mouse models (Jones *et al*, 2007; Kim *et al*, 2007). In prostate epithelial cells, however, re-expression of the IGF1R inhibited the malignant phenotype of SV40 T antigen immortalised human prostate epithelial cells (Plymate *et al*, 1997). Epithelial-specific deletion of *IGF1R* accelerated the emergence of aggressive prostate cancer when p53 activity was compromised (Sutherland *et al*, 2008). These observations underscore the importance of the IGF axis in carcinogenesis and tumour progression and, on the other hand, show again that IGF/INS effects are variable in different cell and tissue types, including cancer.

## IGF1R as a target for cancer therapy

In current clinical trials, the main IGF/INS target for cancer therapy is IGF1R. Other IGF/INS targets are approached indirectly: IGFs are addressed by the use of somatostatin analogues, which suppress their production, and IGF/INSRs through blockage of receptor downstream molecules, using PI3K and AKT inhibitors among other substances. More than 70 clinical trials investigating the blockage of IGF1R are under way. Different anti-IGF1R antibodies, as well as small-molecule IGF1R or IGF1R tyrosine kinase inhibitors, are being investigated as stand-alone therapies or in combination with conventional treatments for many cancers, including breast cancer, colorectal cancer, leukaemia (ALL, CML), non-small-cell lung cancer, ovarian cancer, pancreatic cancer, prostate cancer and sarcoma (recently discussed and summarised in Gualberto and Pollak, 2009). Most anti-IGF1R antibodies are reported to be monospecific for IGF1R, whereas several small-molecule inhibitors also inhibit IGF1R/INSR heterodimers and INSR. Preliminary outcomes have suggested that the treatment is well tolerated, although effects on metabolism, such as elevated levels of circulating IGF1, INS and glucose levels, have been reported. In some cases, anti-diabetic treatment was required to control blood glucose levels (Gualberto and Pollak, 2009). The efficacy of the treatment has not been fully explored thus far, but encouraging data have been registered in a phase-II study concerning the use of an anti-IGF1R antibody in non-small-cell lung cancer. Promising results of anti-IGF1R therapy have also been reported on patients with sarcomas in phase-I studies (Gualberto and Pollak, 2009). These early studies justify the investigation of IGF1R as a target for cancer therapy. However, a phase-III study with an anti-IGF1R antibody combined with erlotinib in advanced non-small-cell lung cancer was terminated recently for safety reasons and lack of efficiency (ClinicalTrials.gov Identifier: NCT00673049).

These results show that the IGF1R is a challenging target for cancer therapy. Clinical trials targeting IGF1R must be designed and controlled with care. Several limitations arise from the complexity and multiple functions of the IGF/INS axis: (a) The IGF/INS axis is a molecular network that exerts essential functions in normal tissues (e.g. control of growth processes, tissue homeostasis, differentiation and metabolism). Interfering with the IGF/INS network may, therefore, cause severe side effects in some tissues and cells. (b) A single anti-IGF1R therapy may be inefficient. Resistance to anti-IGF1R therapy can occur because of signal transduction by IGF/INS heterodimer receptors and redundant effects with INSR. It was recently shown in a mouse model for pancreatic neuroendocrine cancer that resistance to anti-IGF1R therapy was overcome by disruption of the INSR gene (Ulanet *et al*, 2010). Furthermore, mutations in major IGF1R signalling molecules, such as the tumor suppressor PTEN affecting the PI3K–AKT pathway (Zhao *et al*, 2004), and cross-talks with other molecular pathways, such as EGFR (Morgillo *et al*, 2006; Huang *et al*, 2009), can cause anti-IGF1R therapy resistance. (c) A combined therapy targeted against IGF1R and INSR may be more efficient, but will also affect glucose metabolism and provoke diabetic symptoms. A combined therapy targeting IGF1R and EGFR was shown to successfully inhibit tumour cells, which were resistant to IGF1R blockage by overexpressing the EGFR pathway (Huang *et al*, 2009). (d) Although IGF1R was reported to be overexpressed in a variety of cancers, in some tumours the IGF signal exerts a protective effect against tumour formation (Lewis *et al*, 2009) and inhibits the emergence of an aggressive tumour phenotype (Sutherland *et al*, 2008).

Taken together, the data reported thus far suggest that IGF1R is an interesting target in cancer. However, several limitations arise from the complexity and multifunctional role of the IGF/INS axis. To overcome these limitations, a combined therapy focused on IGF1R and other targets, such as EGFR, is a promising treatment strategy for some types of cancer, but the patients must be selected with great care to avoid severe side effects.

## Future directions

Reports from clinical trials give reason for optimism regarding the use of IGF1R inhibitors in cancer patients. At the same time, however, in some trials severe side effects and resistance to anti-IGF1R treatment were observed. To control, predict and – in best case – avoid side effects, we need a better understanding of the function of the IGF/INS pathway in normal physiology, including implications of the IGF/INS axis in normal growth, development, differentiation and cellular metabolism. Furthermore, we need new molecular markers to identify patients profiting of an anti-IGF1R therapy. The clinical success of nearly all tyrosine kinase inhibitors is predicted by appropriate tumour characterisation, such as the presence of mutations or receptor overexpression. An elegant approach to how such a characterisation could be achieved was described by de Bono and coworkers. They confirmed the expression of IGF1R by analysing circulating tumour cells (de Bono *et al*, 2007). Such an analysis – extended to additional molecular markers similar to IGF1R/EGFR expression levels and receptor mutations, as well as alterations of key signalling molecules, such as PTEN, p53 or k-ras – may help to achieve individual tumour characterisation to increase response rates to treatment, reduce side effects and avoid therapy resistance.

## Conclusion

In the IGF/INS axis, IGF and INS proteins functionally cross-talk with each other and form a dynamic mutually interacting multilayer molecular network. This regulatory network exerts multiple functions in normal physiology, but is also implicated in the emergence and progression of cancer. Clinical trials with drugs blocking IGF1R are in progress; their preliminary results have been promising. However, the complexity, multiple functions, and interactions of the IGF/INS axis are limiting factors, which may cause side effects, as well as therapy resistance and pose a challenge for accurate patient selection.

Extensive research in the last few decades has provided important insights into the functions of the IGF/INS axis. Future work should be aimed at a better understanding of the axis as a whole, its role in normal physiology and pathophysiology, and the implications of the manipulation of different components – for example, with the help of systems biology approaches – to establish mechanism-based use of inhibitors for the treatment of cancer and other diseases.
